# Neurite Outgrowth and Neuroprotective Effects of Quercetin from *Caesalpinia mimosoides* Lamk. on Cultured P19-Derived Neurons

**DOI:** 10.1155/2013/838051

**Published:** 2013-06-11

**Authors:** Napat Tangsaengvit, Worawan Kitphati, Sarin Tadtong, Nuntavan Bunyapraphatsara, Veena Nukoolkarn

**Affiliations:** ^1^Department of Pharmacognosy, Faculty of Pharmacy, Mahidol University, Bangkok 10400, Thailand; ^2^Department of Physiology, Faculty of Pharmacy, Mahidol University, Bangkok 10400, Thailand; ^3^Faculty of Pharmacy, Srinakharinwirot University, Ongkharak, Nakhon Nayok 26120, Thailand

## Abstract

Quercetin has been isolated for the first time from ethyl acetate extract of *Caesalpinia mimosoides* Lamk. * C. mimosoides* Lamk. (Fabaceae) or Cha rueat (Thai name) is an indigenous plant found in mixed deciduous forest in northern and north-eastern parts of Thailand. Thai rural people consume its young shoots and leaves as a fresh vegetable, as well as it is used for medicinal purposes.The antioxidant capacity in terms of radical scavenging activity of quercetin was determined as IC_50_ of 3.18 ± 0.07 *µ*g/mL, which was higher than that of Trolox and ascorbic acid (12.54 ± 0.89 and 10.52 ± 0.48 *µ*g/mL, resp.). The suppressive effect of quercetin on both purified and cellular acetylcholinesterase (AChE) enzymes was investigated as IC_50_ 56.84 ± 2.64 and 36.60 ± 2.78 *µ*g/mL, respectively. In order to further investigate the protective ability of quercetin on neuronal cells, P19-derived neurons were used as a neuronal model in this study. As a result, quercetin at a very low dose of 1 nM enhanced survival and induced neurite outgrowth of P19-derived neurons. Furthermore, this flavonoid also possessed significant protection against oxidative stress induced by serum deprivation. Altogether, these findings suggest that quercetin is a multifunctional compound and promising valuable drugs candidate for the treatment of neurodegenerative disease.

## 1. Introduction

*Caesalpinia mimosoides* Lamk., a small spiny tropical trees or climbing shrubs belonging to family Fabaceae (subfamily: Caesalpinioideae), is mainly distributed in the south of China and grows in countries like India, Myanmar, as well as in northern and north-eastern parts of Thailand [1, 2]. Young sprouts and leaves are edible and sour and are traditionally used as a carminative and a remedy for dizziness [1]. In addition, the folk practitioners of Udupi district of India used the roots for ulcer and wound management, as well as for the treatment of arthritis [2]. Furthermore, this plant showed *in vivo* antiarthritic and analgesic activities [2]. The methanolic extract of *C. mimosoides* shoot tips was reported to exhibit antioxidant activity [3]. Moreover, the aqueous and the ethanol extracts contained gallic acid, the antioxidative compound [1]. Based on these data, we postulated that this plant may compose of constituents that could exert some neuroprotective effects analogous to that of antioxidants.

 Alzheimer's diseases (AD), a neurodegenerative disease, is the most common cause of senile dementia adversely affecting a significant proportion of the world's population. AD is characterized clinically by the progressive and irreversible loss of neurons in the brain [4–8]. On the other hand, brain amyloid-*β* plaques and neurofibrillary tangles, in addition to loss of neurons and their synaptic projections, are the main pathological features of AD [4–8]. Though the onset of this disease is still unclear, many studies have provided evidence for the deleterious consequences of oxidative stress (OS), which plays a significant causative role in the disease process [5–9]. So far, one of the most promising approaches for treating this disease is to enhance the acetylcholine (ACh) level in the brain using AChE inhibitors which merely provide short-lived symptomatic relief. More recently, therapeutic approach, in which drug candidates are designed to possess diverse pharmacological properties and act on multiple targets, has stimulated the development of the multifunctional drugs [7–11].

 On the other hand, quercetin (3,3′,4′,5,7-pentahydroxyflavone) is the major representative flavonol-type flavonoid found in various plants including fruits, vegetables, tea, wine, and honey [12, 13]. Recently, quercetin has been marketed in the United States primarily as a dietary supplement [14]. Quercetin is a well-known potent antioxidant, which could be a result of scavenging of radicals, metal chelation, enzyme inhibition, and/or induction of the expression of protective enzymes [12, 13]. Accordingly, OS and production of free radicals tend to increase with age, whereas the body's natural antioxidant defenses decline. Cell damage caused by OS is thought to contribute to the development of certain disorders such as cancer and neurodegenerative disorders, including AD [6–9]. Particularly, oxidative damage is the most marked in the brain due to its high oxygen consumption, high fatty acids levels, and low antioxidant enzyme levels. Moreover, neurons are largely postmitotic, so they cannot be replaced readily via mitosis when damaged [15, 16]. Thus, the antioxidant properties certainly contribute to their neuroprotective effects. To combat with such complex disease like AD, the search for multifunctional agents that simultaneously possess anticholinesterase, antioxidant, and neuroprotection activities emerges as a new strategy for the development of new drugs. In this study, quercetin, an active compound from *C. mimosoides,* was investigated for antioxidant, anti-AChE, and neuroprotective effects. In order to further investigate the benefit of quercetin on neuronal cells, P19-derived neurons were used as a neuronal model in this study.

## 2. Materials and Methods

### 2.1. Plant Material

The young sprouts and leaves of *C. mimosoides* Lamk. were collected from Maha Sarakham Province, then authenticated by Professor Dr. Wongsatit Chuakul, Faculty of Pharmacy, Mahidol University, Thailand. A voucher specimen (BKF number 173175) was deposited in the herbarium, Royal Forest Department, Bangkok, Thailand. The plant material was washed thoroughly and dried in a hot air oven at 40°C before ground to a fine powder.

### 2.2. Chemicals and Enzymes

Acetylthiocholine iodide (ATCI), lyophilized powder of AChE (a purified enzyme from eel (*Electrophorus electricus*) type VI-s, 425.94 units/mg, 687 U/mg protein), 5,5-dithiobis [2-nitrobenzoic acid] (DTNB), galanthamine, and bovine serum albumin (BSA) were obtained from Sigma (St. Louis, MO, USA). Alpha minimal essential medium (*α*-MEM), newborn calf serum (NCS), fetal bovine serum (FBS), and antimycotic solution were purchased from Gibco, USA. All transretinoic acids (RAs), cytosine-1-*β*-D-arabinoside (Ara-C), 1 : 250 porcine trypsin, poly-L-lysine (MW > 300,000), dimethyl sulfoxide (DMSO), XTT (sodium 2,3,-bis(2-methoxy-4-nitro-5-sulfophenyl)-5-[(phenylamino)-carbonyl]-2H-tetrazolium inner salt), phenazine methosulphate (PMS), and phosphate buffer saline (PBS) were obtained from Sigma, USA. Analytical grade methanol (MeOH) was purchased from Merck, Germany. T-25 flask, 6-well, and 96-well plates were purchased from Corning, USA. Bacteriological grade culture dishes were obtained from Hycon Plastic Inc., USA.

### 2.3. Extraction and Isolation

Powdered plant material was macerated with hexane, ethyl acetate (EtOAc), and MeOH, respectively. The extracts were filtered and then evaporated to dryness under reduced pressure at 40°C and screened for antioxidant activity. The active EtOAc extract (45 g) was chromatographed on flash column packed with silica gel (grade 7734, 70–230 mesh) and eluted with hexane and increasing polarities with EtOAc and MeOH, respectively, to yield 8 fractions. Fraction F7 (1.20 g), the active fraction eluted with 50–80% EtOAc hexane, was recrystallized and further purified on a Sephadex LH-20 column using 100% MeOH as eluent to give compound **1** (518 mg) and compound **2** (24 mg).

### 2.4. Determination of Antioxidant Activity: DPPH Microplate Assay

The antioxidant capacity was estimated in terms of radical scavenging activity according to a modified version of Brand-Williams method [17]. Briefly, 100 *μ*L of tested compounds at least 5 concentrations (dissolved in MeOH) was thoroughly mixed with 100 *μ*L of freshly prepared DPPH solution (3 × 10^−5^ M dissolved in MeOH). The reaction mixture was incubated for 30 min, then, the absorbance was read at 517 nm. Each assay was done in triplicate. IC_50_ value was determined by a linear regression analysis between the inhibition percentages against the concentration of tested compounds by using the Excel program.

### 2.5. Determination of Anticholinesterase Activity: Microplate Assay

Ellman's colorimetric method [18, 19] and modified method using 96-well microplates [20] were used. Briefly, acetylthiol, the product of hydrolysis reaction of ACh by cholinesterase, will react with DTNB to give 2-nitro-5-thiobenzoate (NTB^−^), which ionizes to the NTB^2-^ dianion (in water at pH 8). This NTB^2-^ ion appears as yellow, which is quantified by measuring the absorbance of visible light at 405 nm. Briefly, tested compound was prepared in buffer a containing 50% MeOH. In a 96-well microtiter plate, 25 *μ*L of tested sample was added to 200 *μ*L reaction medium that consisted of 50 *μ*L of buffer (50 mM TrisHCl pH 8.0) containing 0.1% BSA, 125 *μ*L of 3 mM DTNB in buffer containing 0.1 M NaCl, and 0.02 M MgCl_2_·2H_2_O and 25 *μ*L of 15 mM ATCI in deionized water. These contents were mixed and preincubated for 5 min at 37°C. The plate was pre-read at 405 nm using a microplate reader (TECAN M200, Switzerland). Thereafter, the reaction was initiated by the addition of 25 *μ*L of AChE (0.22 U/mL). After 20 min incubation at 37°C, absorbance was measured again within 4–7 min. The reaction control consisted of reaction medium (200 *μ*L), 25 *μ*L of enzyme, and 25 *μ*L of 50% MeOH in buffer. Two blanks were also carried out (with and without sample), and MeOH was used as the solvent control. MeOH was controlled at 5% of the final volume, since it had been found that in this proportion it does not affect the AChE activity as well as thiols determination and the degree of AChE inhibition [21]. Galanthamine (50% MeOH) served as the positive control. Each assay was done in triplicate.

### 2.6. Determination of Anticholinesterase Activity in Neuroblastoma Cells

#### 2.6.1. Cell Culture

The human neuroblastoma cell line SK-N-SH was purchased from the American Type Culture Collection (Manassas, VA, USA). The SK-N-SH cells were cultured in minimum essential medium from Gibco (California, USA), supplemented with 10% heat inactivated FBS, 2% supplementary amino acid solution, and 1% Glutamax (Gibco, California, USA), 1% penicillin/streptomycin. Incubation was carried out at 37°C in a humidified atmosphere of 5% CO_2_-95% O_2_ atmosphere. Cells were seeded into 96-well microplates (Nunc, Roskilde, Denmark) at a density of 1 × 105 cells/mL (100 *μ*L in each well). Experiments were carried out after 24 h of seeding. 

### 2.7. Measurement of Anticholinesterase Activity on Cellular AChE

 One of the important neuronal properties of SK-N-SH cells is the synthesis of neurotransmitter enzymes [22, 23]. The inhibition activity of samples on this cell line was investigated by the modified Ellman's colorimetric method [17, 18]. The condition was slightly modified to enable the cellular enzyme to work properly [19]. Briefly, 25 *μ*L of sample dissolved in buffer containing 50% methanol, 100 *μ*L of 3 mM DTNB in buffer containing 0.1 M NaCl and 0.02 M MgCl_2_·2H_2_O, and 25 *μ*L of 15 mM ATCI in deionized water were added to the well containing 100 *μ*L of the cells. The absorbance at 405 nm was measured by a microplate reader (TECAN M200, Switzerland). All assays were done in triplicate.

### 2.8. Neuroprotective Activity on P19 Embryonic Carcinoma Cells

#### 2.8.1. Cell Culture

Murine P19 embryonic carcinoma cells were purchased from American Type Culture Collection (ATCC), USA. P19 cells were cultured as described by Mcburney [24, 25] in a slightly modification [26, 27]. In brief, the undifferentiated P19 cells were cultured as monolayers in *α*-MEM with 7.5% NCS, 2.5% FBS, and 1% antibiotics-antimycotic solution in a 25-cm^2^ tissue culture flask, incubated at 37°C in a humidified atmosphere of 5% CO_2_. Cells in monolayer cultures were maintained in exponential growth phase by subculturing every 2 days until use.

### 2.9. Neuronal Differentiation of P19 Cells

 The exponentially grown cultures indicated the cells density by trypan blue exclusion assay. Then, neuronal differentiation was induced by seeding 2 × 10^6^ cells/mL cell in a 100 mm bacteriological grade culture dish containing 10 mL *α*-MEM supplemented with 5% FBS, 1% antibiotics-antimycotic solution, and 0.5 *μ*M RA. Under these conditions, cells did not adhere to dishes but instead formed large aggregates in suspension (neurospheres). After 4 days of RA treatment, aggregates were dissociated with trypsin, washed, resuspended on poly-L-lysine-precoated 96-well plates (plates were previously coated with 50 *μ*g/mL poly-L-lysine dissolved in PBS for overnight and sterilized under UV light for 30 min) at a cell density of 7 × 10^4^ cells/mL (150 *μ*L/well) in *α*-MEM supplemented with 10% FBS and 1% antibiotics-antimycotic solution, and incubated for 24 h. Ara-C (10 *μ*M) was added at day one after plating to inhibit the proliferation of nonneuronal cells. The medium was changed every 2-3 days. The differentiated P19-derived neurons were used after day 14 of the differentiation process.

### 2.10. Evaluation of Cell Viability (Measurement of Cell Density)

 The trypan blue exclusion assay was based on the capability of viable cells to exclude the dye. Because viable P19 cells maintained membrane integrity, the cells did not allow trypan blue dye to pass through the cell membrane. Only cells with damaged membrane appeared blue because their accumulations of dye were counted as dead [28]. 

 The embryo body (aggregated neuron cells), which is treated with RA in Petri dishes, was collected into a centrifuge tube. Samples were centrifuged at 1500 rpm for 20 min and the temperature was controlled at 25°C; the supernatant was gently removed and the cell pellet was then resuspended. The aggregated cells were rewashed again two times, the medium was changed and centrifuged at 1500 rpm for 5 min, and then the precipitate was resuspended in corresponding medium. The cell suspension was stained with equal volume of 0.2% trypan blue in PBS, incubated at room temperature for 3 min, and loaded into a hemocytometer. The viable cells were counted under an inverted microscope, and the cell density was calculated.

### 2.11. XTT Reduction Assay for Neuronal Cells Viability

 The procedure was carried out on P19-derived neurons cultured in a 96-well plate [29]. After 14 days of differentiation process, the *α*-MEM supplemented with 10% FBS, 10 *μ*M Ara-C, and 1% antibiotics-antimycotic solution was removed and replaced with DMSO solutions of samples, diluted with the *α*-MEM supplemented with 10% FBS, and 1% antibiotics-antimycotic solution in the presence of 10 *μ*M Ara-C was added to give the concentrations of 0.0001, 0.001, 0.01, 0.1, 1, and 10 *μ*M. The final DMSO concentration on the assay was kept at 0.5% which had no effect on cell viability. The blank control wells contained the corresponding medium (*α*-MEM supplemented with 10% FBS, 10 *μ*M Ara-C, and 1% antibiotics-antimycotic solution). The cells were incubated for 18 h at 37°C. Then 150 *μ*L of the medium was removed, and after that 50 *μ*L of XTT reaction solution (12 mL of XTT 1 mg/mL in *α*-MEM, 30 *μ*L of PMS (an intermediate electron acceptor), and 10 mM in PBS were added. Blank control was performed by adding XTT reaction solution without cells. Shake the plate gently to evenly distribute the dye in the wells. After incubated at 37°C for 4 hours, 150 *μ*L of PBS was added to each well. The optical density (OD) value was determined on a microplate reader at wavelength of 450 nm. Absorbance values that were higher than control conditions indicate an increase in cell viability. The data were expressed as the mean ± SEM (*n* = 3), with the medium as a control representing 100% cell viability. The concentration that enhanced survival of cultured neurons more than control will be further investigated for neuritogenic and neuroprotective activity against OS induced by serum deprivation.

### 2.12. Neuritogenic Assay

 The assay was carried out with P19-derived neurons cultured in a poly-L-lysine-precoated 6-well plate [26]. After 14 days of differentiation process, the *α*-MEM supplemented with 10% FBS, 10 *μ*M Ara-C, and 1% antibiotics-antimycotic solution was removed, and DMSO solution of quercetin, diluted with the *α*-MEM supplemented with 10% FBS, 10 *μ*M Ara-C, and 1% antibiotics-antimycotic solution, was added. The concentration of DMSO was added to the cultures at 0.5%. The *α*-MEM supplemented with 10% FBS, 10 *μ*M Ara-C, and 1% antibiotics-antimycotic solution was added into control wells. The cells were incubated for 24 h at 37°C in a humidified atmosphere of 5% CO_2_. Geldanamycin 1 nM was used as positive control. The morphology under a phase-contrast microscope was observed. The appearance of P19-derived neurons was compared to the control (vehicle without quercetin) and measured for the length and number of neurites. Average length and number of neurites of 30 neurons from the assay were measured. The assay was performed in a replicate. The data were expressed as the mean ± SEM from three independent experiments.

### 2.13. Neuroprotective Activity against OS Induced by Serum Deprivation

 For serum withdrawal-induced oxidative stress [27], cells were seeded and cultured in the *α*-MEM supplemented with 10% fetal bovine serum for 24 h, washed with *α*-MEM three times, and cultured in serum-free *α*-MEM (*α*-MEM plus 10 *μ*M Ara-C) in the absence or the presence of quercetin for 18 h. The blank control was cultured in *α*-MEM supplemented with 10% FBS and 10 *μ*M Ara-C. The cell survival ability was measured using the XTT method.

## 3. Results

### 3.1. Extraction and Isolation

**Figure 1:**
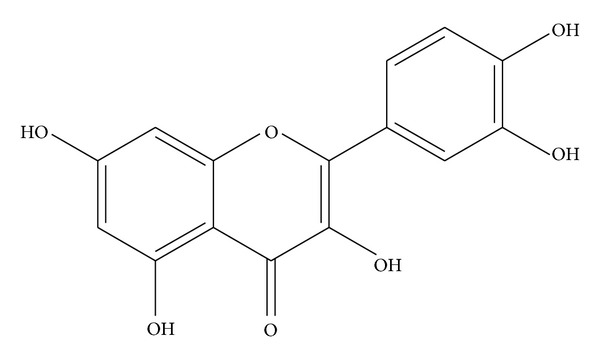
Chemical structure of quercetin.

The yields of hexane, ethyl acetate, and methanol extracts were 1.35, 4.72, and 17.50% w/w, respectively. Bioactivity-guided isolation of the active compounds from the EtOAc extract which exhibited potent DPPH radical scavenging activity led to the isolation of two known compounds. Through the comparison of the physical property and spectroscopic data comparing with the literature values [30–32], the isolated compounds were identified as gallic acid (**1**) and quercetin (**2**, Figure 1). This is the first report on the isolation of quercetin from this plant. 

*Gallic acid* (**1**). white crystal, mp 253–256°C; IR (KBr): 3492, 3368, 3288, 1703, 1619, 1541, 1450, 1247, 1027 cm^−1^; ^1^H NMR (300 MHz, DMSO-*d*_6_) ***δ**_H_* 6.90 (2H, s, H-2 and H-7); ^13^C NMR (75 MHz, DMSO-*d*_6_) ***δ**_C_* 108.9 (C-3 and C-7), 120.7 (C-2), 138.3 (C-5), 145.6 (C-4 and C-6), 167.7 (C-1); ESI-MS *m/z* 169.20 [M-H]^−^. 

*Quercetin* (**2**). yellow crystal, mp 318–320°C; IR (KBr): 3282. 1743, 1666, 1610, 1517, 1430, 1211, 1094 cm^−1^; ^1^H NMR (300 MHz, DMSO-*d*_6_) ***δ**_H_* 6.14 (1H, d, *J *= 2.0, H-6), 6.36 (1H, d, *J *= 2.0, H-8), 7.65 (1H, d,* J *= 2.0 H-2′), 6.87 (1H, *J *= 9.0 Hz, H-5′), 7.53 (1H, dd, *J *= 2.0, 9.0, Hz, H-6′); ^13^C NMR (75 MHz, DMSO-*d*_6_) ***δ**_C_* 148.6 (C-2), 137.2 (C-3), 176.6 (C-4), 161.5 (C-5), 99.2 (C-6), 165.5 (C-7), 94.3 (C-8), 157.0 (C-9), 103.6 (C-10), 122.8 (C-1′), 116.4 (C-2′), 146.0 (C-3′), 153.4 (C-4′), 115.8 (C-5′), 120.8 (C-6′); ESI-MS *m/z* 301.39 [M-H]^−^.

### 3.2. Free Radical Scavenging Property

 The antioxidant activity was evaluated in terms of radical scavenging property. Quercetin and gallic acid strongly possessed antioxidant activity with IC_50_ of 3.18 ± 0.07 and 4.83 ± 0.03 *μ*g/mL, respectively, which were stronger than that of Trolox and ascorbic acid (12.54 ± 0.89 and 10.52 ± 0.48 *μ*g/mL, resp.).

### 3.3. Anticholinesterase Activity

Quercetin and gallic acid possessed anticholinesterase activity both upon purified and cellular AChE with respective IC_50_ values of 56.84 ± 2.64 and 36.60 ± 2.78 *μ*g/mL for quercetin, and 12.73 ± 0.56 and 2.97 ± 0.17 *μ*g/mL for gallic aid, whereas IC_50_ of galanthamine (positive control) was 1.73 ± 0.12 *μ*g/mL and 0.23 ± 0.02 *μ*g/mL, respectively, (Table 1).

### 3.4. Effects on P19-Derived Neuron Viability

Two isolated compounds, gallic acid and quercetin, which possessed potent antioxidant and anticholinesterase activity, were selected to determine their neuroprotective capacity. The P19 cells, isolated from an experimental embryo-derived teratocarcinoma in mice, are widely used as *in vitro* model because of their specific characteristics. Exposing aggregated P19 cells to retinoic acid (RA) effectively induces the development of neurons, astroglia, and microglia cell types [24–27]. Unlike P12 cell line, P19-derived neurons are irreversibly postmitotic; moreover, these neurons exhibit many characteristics of mature CNS neurons containing particular neurotransmitters such as *γ*-aminobutyric acid (GABA) and acetylcholine [25–27].

 The biological effects on P19-derived neurons of two compounds at a serial of dilutions in a microplate were identified and quantified; the survival of cells was determined by using a XTT reduction assay. Viable cells with active mitochondrial dehydrogenase caused cleavage of the tetrazolium ring into a visible red-orange product through a formazan reaction, while dead cells remained a light orange color of which the OD was measured in a microplate reader at 450 nm. After 24 h of quercetin treatment, the result indicated that a very low dose of 1 nM quercetin promoted high cell viability of cultured neurons (% cell viability > 100%) more than control without any cytotoxicity (IC_50_ > 10 *μ*M), while gallic acid was found to be cytotoxic to the cell. Therefore, only quercetin was chosen for further evaluation of neuritogenic and neuroprotective activity.

### 3.5. Neuritogenic Activity

**Figure 2:**
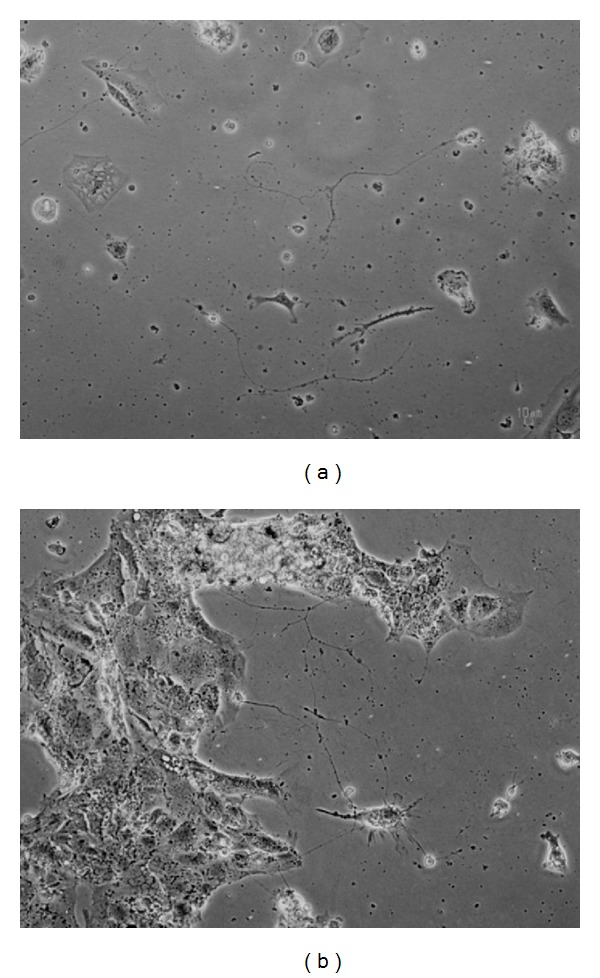
Effect of quercetin on neurite outgrowth from P19-derived neurons. Cells were treated without or with quercetin 1 nM for 24 h. Phase-contrast micrographs of control (a) and treatment with quercetin (b); scale bar, 10 *μ*m.

 The characterization of neurite formation, maturation, and collapse/resorption is an area of intense interest; particularly, it is a readjustment in the normal neuronal functions and local circuits in the damaged CNS. Measurement of the length of outgrowth per cell is the most commonly used to assess the ability of a compound that affects the growth of neurite [33, 34]. P19-derived neurons grown in a poly-L-lysine-precoated 6-well plate were treated with or without 1 nM quercetin for 24 h. The morphology of 30 neuronal cells was examined using phase-contrast micrographs. The result showed that neurite outgrowth was induced in P19-derived neurons by quercetin compared with an active control, geldanamycin. Quercetin not only significantly increased the amount of neurites (3.23 ± 1.55), but also the neurite length (139.00 ± 108.80 *μ*m) (Figure 2), whereas geldanamycin exhibited the amount and length of neurite at 2.03 ± 1.30 and 3.23 ± 1.55 *μ*m, respectively.

### 3.6. Neuroprotective Activity against OS Induced by Serum Deprivation

**Figure 3:**
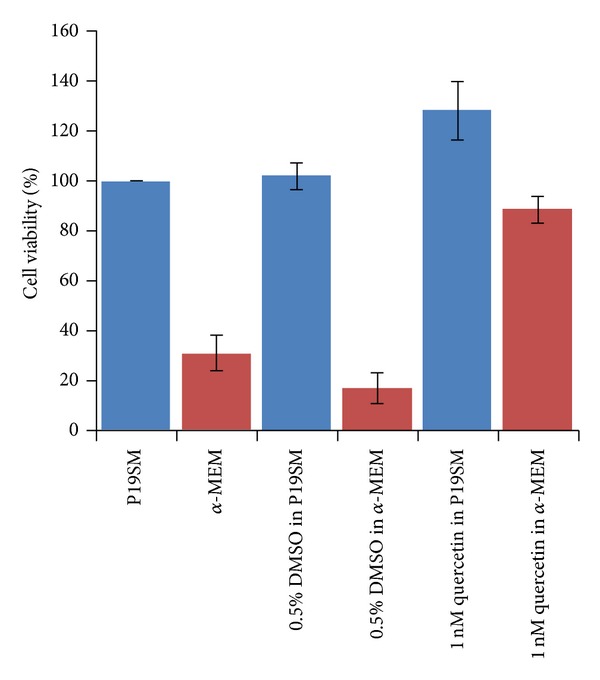
The effect of quercetin in neuronally differentiated P19 cells against oxidative stress induced by serum deprivation. The histogram shows the percentage of cell viability relative to vehicle-treated control cultures. Each bar represents mean ± SEM from three individual measurements.

 To determine whether quercetin protects neurons from oxidative stress and induces cell death triggered by serum deprivation, six experiments were performed, cultured in serum-free or complete medium, alone or with 1 nM quercetin. After 24 h, the amount of survival cells in each experiment was measured by XTT assay and typical histograms were shown in Figure 3. A dramatic decrease in cell viability was observed, in which P19 neuronal cells were cultured in *α*-MEM. Serum deprivation reduced the cell survival to 31.16% as compared to the untreated control (P19SM). However, pretreatment of P19 neuronal cells with 1 nM quercetin was effective in increasing the survival in both serum-supplemented and serum-deprived cultures up to 128.01 and 88.29% as compared to the control and *α*-MEM-treated cell, respectively.

## 4. Discussion

From the present study, quercetin was isolated for the first time from the EtOAc extract of *C. mimosoides*. The strong antioxidant activity of quercetin was evaluated in terms of free radical scavenging capacity which was more potent than Trolox and ascorbic acid. Quercetin also exerted cholinesterase inhibitory activity on both purified and cellular AChE enzymes. Furthermore, quercetin at a very low dose of 1 nM enhanced the survival of P19-derived neurons, significantly increased neurite outgrowth, as well as showed powerful neuroprotective action against OS induced by serum deprivation. Since the damaged brain lacks the reconstructive capacity, the use of compounds that are capable of enhancing the action of neurotrophic factors to stimulate neurite outgrowth seems to be an important step in the process of neuronal regeneration [34]. To assess neuritogenicity of quercetin, we quantified the neurite outgrowth from P19-derived neurons which is dependent on microtubule formation and a key to restoring proper function. Our result showed that quercetin produced a significant neurite outgrowth from P19-derived neurons.

Considerately, serum is a mixture that consists of hundreds of proteins and some vital growth factors needed for proliferation of cells in culture which functions as hormonal factors that stimulate cell growth transport proteins that carry hormones, lipids, minerals, and trace elements, and stabilizing and detoxifying factors [35]. Mounting evidence suggests that serum deprivation induced oxidative stress due to a lack of necessary nutrients and trophic factors, triggered mitochondrial ROS generation, with activation of the intrinsic (caspase 9-dependent) apoptotic pathway and release of cytochrome *c*, resulting in cell apoptosis [36, 37]. An increasing number of studies have also revealed that drugs or other therapeutics can prevent serum deprivation-trigger cell death by scavenging the intracellular ROS or implicating it in apoptosis signaling under those conditions [36]. Our examination found that quercetin at a very low dose of 1 nM effectively reduced neuronal cell death caused by serum deprivation-triggered OS, comparison to its antioxidant activity that exhibiting IC_50_ in micromolar range (9.4 *μ*M). These observations imply that the neuroprotective effect of quercetin may act as a modulator of cell signaling, not an antioxidant. However, further studies are also required to understand the mechanism of its neuroprotective action.

## 5. Conclusion

The present study described the isolation of quercetin from* C. mimosoides* Lamk. The results indicated that quercetin, formerly thought to be a radical scavenger, is now considered as an anticholinesterase as well as a significant neuroprotective agent. In view of its multiple biological activities, quercetin holds a great promise as a potential therapeutic agent for Alzheimer's diseases and other neurodegenerative diseases. 

## Figures and Tables

**Table 1:** Antioxidant and anticholinesterase activities of isolated compounds.

	Antioxidant		Anticholinesterase (using purified enzyme from *E. electricus*)		Anticholinesterase (using neuroblastoma cell)	
	% inhibition (at 100 *µ*g/mL)	IC_50_ (*µ*g/mL)	% inhibition (at 100 *µ*g/mL)	IC_50_ (*µ*g/mL)	% inhibition (at 100 *µ*g/mL)	IC_50_ (*µ*g/mL)
Gallic acid	94.76 ± 0.51	4.83 ± 0.02	73.64 ± 0.06	12.73 ± 0.56	90.67 ± 2.66	2.79 ± 0.17

Quercetin	92.61 ± 0.99	3.18 ± 0.07	80.94 ± 2.52	56.84 ± 2.64	82.0 ± 3.34	36.60 ± 2.78

Positive control	Trolox: IC_50_ 12.54 ± 0.89 Ascorbic acid: IC_50_ 10.52 ± 0.48		Galanthamine IC_50_ 0.81 ± 0.04		Galanthamine IC_50_ 0.23 ± 0.02	

Values are mean ± SEM (*n* = 3).
